# Abscopal Effect Following Proton Beam Radiotherapy in a Patient With Inoperable Metastatic Retroperitoneal Sarcoma

**DOI:** 10.3389/fonc.2019.00922

**Published:** 2019-09-26

**Authors:** Randall J. Brenneman, Nima Sharifai, Benjamin Fischer-Valuck, Comron Hassanzadeh, Jeffrey Guzelian, John S. A. Chrisinger, Jeff M. Michalski, Peter Oppelt, Brian C. Baumann

**Affiliations:** ^1^Department of Radiation Oncology, Washington University School of Medicine, St. Louis, MO, United States; ^2^Department of Pathology and Immunology, Washington University School of Medicine, St. Louis, MO, United States; ^3^Department of Radiation Oncology, Winship Cancer Institute, Emory University, Atlanta, GA, United States; ^4^Division of Nuclear Medicine, Mallinckrodt Institute of Radiology, Washington University School of Medicine, St. Louis, MO, United States; ^5^Division of Medical Oncology, Department of Medicine, Washington University School of Medicine, St. Louis, MO, United States

**Keywords:** retroperitoneal sarcoma, abscopal effect, proton therapy, metastasis, SMARCB1

## Abstract

**Background:** Retroperitoneal sarcomas (RPS) are rare and primarily managed with surgery, which improves local recurrence-free and overall survival. Radiation can improve local control or provide palliation for inoperable or metastatic RPS by eliciting tumor cell death via irreparable DNA damage. In extraordinary circumstances radiation-induced cell death promotes immune-mediated regression of non-irradiated lesions in a process termed the abscopal effect. Abscopal effects are rare and incompletely understood, involving a balance of radiation's immunogenic and immunosuppressive effects. There are currently no methods to predict abscopal responses following radiotherapy. Case reports documenting post-radiotherapy abscopal effects provide additional information to better characterize these responses and to inform ongoing and future clinical trials attempting to harness radiation-induced immune responses to improve outcomes with systemic therapy, such as SARC-032, a cooperative group trial of pre-operative radiation ± pembrolizumab. We present a case of inoperable metastatic RPS treated with proton radiotherapy with complete responses of un-irradiated metastases.

**Case Presentation:** A 67 year-old female with inoperable metastatic unclassified round cell RPS was treated with palliative proton radiotherapy only to the primary tumor. Following completion of radiotherapy, the patient demonstrated complete regression of all un-irradiated metastases, and near complete response of the primary lesion without additional therapy.

**Conclusions:** Metastatic RPS is typically managed with first-line chemotherapy, with objective response rates <50%. We present a case of inoperable metastatic RPS treated with palliative proton radiotherapy for rapidly progressive disease who had complete regression of non-irradiated metastases consistent with the abscopal effect. To our knowledge this is the first case report describing abscopal effects in inoperable metastatic RPS treated with proton radiation and is among the first case reports of an abscopal effect in a patient treated with proton therapy regardless of disease site. Further investigation is warranted regarding the benefit of proton radiation to primary tumors for inoperable metastatic RPS.

## Background

Retroperitoneal sarcomas (RPS) are rare cancers of mesenchymal origin encompassing numerous subtypes, representing 15% of soft-tissue sarcomas (STS) that comprise 1–2% of all cancers (1, 2). RPS are typically well-differentiated/dedifferentiated liposarcoma (WDLS, DDLS) or leiomyosarcoma (LMS), with <10% considered unclassified (3–6). Histology and stage at presentation predict treatment response and recurrence patterns, with poorer outcomes for higher grade, unclassified/less differentiated histologies, and incompletely resected disease (6). Patients often present at advanced stages due to asymptomatic growth in the retroperitoneal space. Definitive management of RPS is based on retrospective data and includes primary resection to negative margins at high-volume centers to improve local recurrence-free and overall survival (OS) (3, 4, 7, 8). Retrospective data indicates that perioperative radiation improves OS, with higher toxicity post-operatively; however, recent preliminary data from the STRASS (Surgery With or Without Radiation Therapy in Untreated Nonmetastatic Retroperitoneal Sarcoma, EORTC 62092) trial failed to demonstrate a recurrence-free survival benefit for pre-operative radiation, although patient numbers on this trial were fairly modest (9, 10). Multimodality RPS treatment yields 5-year OS ranging from 40 to 70% depending on the grade, extent of resection, histological subtype, and perioperative treatment. Local recurrence is common, occurring in 50% of patients by 5 years and is associated with significant morbidity and mortality, hence the importance of local control (2–6, 11–13).

Patients with RPS and metastases at presentation represent 10–20% of RPS cases and have poor outcomes, with median survival of 16 months and 5% 5-year OS (13). Systemic treatment using anthracycline-based regimens improves OS in metastatic patients; however, objective response rates are <20% with few complete responses, and variable responses dependent on tumor histology (13). Surgery for primary and metastatic lesions may improve OS, however data is limited (3). The METASARC study was a retrospective observational analysis of treatment patterns and outcomes for of over 2,000 patients with metastatic STS across multiple histologies (14). At 5 years post-treatment over 80% of surviving patients received locoregional treatment of metastatic lesions, including surgery, radiation, or radiofrequency ablation, with an odds ratio for survival of 7.41 that remained significant on multivariate analysis (14). Approximately 75% of patients in METASARC received first-line polychemotherapeutic regimens containing doxorubicin, with median OS dependent on histology, ranging from 11.0 months for undifferentiated pleomorphic sarcoma to 24.9 months for LMS (14). The Trans-Atlantic RPS Working Group (TARPSWG) recently issued a consensus approach for metastatic RPS recommending anthracycline-based chemotherapy for first-line management given success in extremity STS (15). These guidelines specifically exclude rarer RPS histologies [e.g., rhabdomyosarcoma, unclassified sarcomas, Ewing sarcoma (EWS)/EWS fusion gene-negative small round blue cell sarcomas] making it difficult to provide treatment recommendations for etiologies besides WDLS/DDLS/LMS (15).

Radiotherapy is rarely recommended for first-line treatment of metastatic RPS because systemic therapy can treat both primary and disseminated lesions. Radiation induces significant gastrointestinal toxicity when treating larger lesions to curative doses due to the close proximity of small bowel to the retroperitoneal space. Instead, radiotherapy is typically used for palliation or consolidation following systemic therapy. Sarcoma histology may influence radio-responsiveness, with observed response rates <50% for some rarer variants; however, this observation is limited by small sample sizes (16). Sarcomas typically metastasize via hematologic spread to the lungs or liver. Isolated lymph node metastases occur in only 2–4% of metastatic RPS patients at presentation and carry a poor prognosis, with an estimated median survival of 12.8 months (17, 18). Radiation to metastatic sarcoma can be beneficial, as stereotactic body radiation therapy for sarcoma pulmonary oligometastases is an accepted option in the 2019 NCCN guidelines based on results from the Penn experience (19). However, there are no clinical series describing a standard approach for primary radiotherapy without neoadjuvant/adjuvant treatment for RPS with lymph node metastases due to the rarity of this presentation and widespread use of first-line systemic agents.

## Case Presentation

**Figure 1:**
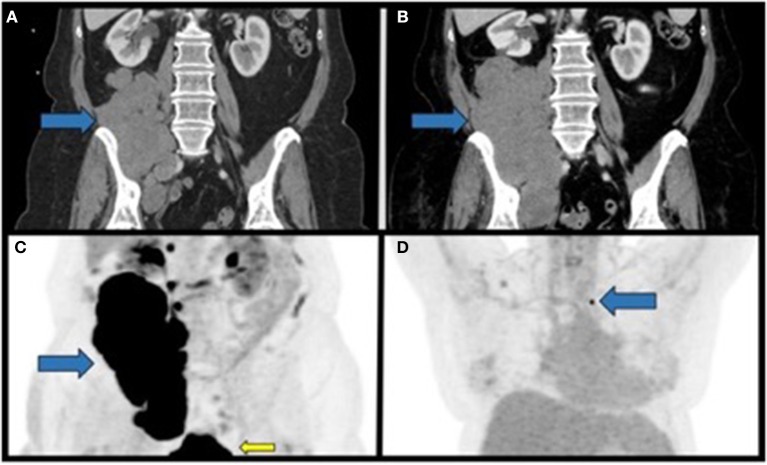
Staging CT scans and PET imaging of the primary tumor and metastatic lesion prior to radiation therapy. **(A)** Coronal section of the initial CT of the abdomen/pelvis with contrast identifying a large soft-tissue mass in the right retroperitoneal space overlying the right psoas muscle with extension into the right hemi-pelvis. **(B)** Coronal section of a CT of the abdomen/pelvis approximately 2 months after the initial diagnosis demonstrating interval growth of the now inoperable retroperitoneal mass. **(C)** Coronal PET/CT maximum intensity projection (MIP) performed approximately 3 months after initial diagnosis, demonstrating the large FDG-avid mass in the right retroperitoneal space (blue arrow). FDG-avidity in the inferior aspect of the image in the figure corresponds to physiological uptake in the urinary bladder (yellow arrow). **(D)** Coronal PET/CT MIP from the same study as in C demonstrating a non-enlarged FDG-avid lymph node in the left supraclavicular nodal station. Blue arrows, primary mass; yellow arrow, physiologic FDG uptake.

A 67 year-old Caucasian female with no prior oncologic history presented to a local hospital with a multi-week history of right lower extremity edema and pain. CT abdomen/pelvis identified a 17.5 × 8.8 × 5.2 cm lobulated retroperitoneal soft tissue mass extending into the right pelvis without evidence of metastases (Figure 1A). CT-guided core needle biopsy demonstrated a high-grade, poorly differentiated neoplasm (Supplemental Figure 1). Given concern for primary RPS she was referred to our institution for further workup.

She was evaluated by surgical oncology and discussed at a multidisciplinary sarcoma tumor board that recommended primary resection if staging scans were negative for metastatic disease. CT chest/abdomen/pelvis identified primary mass progression to 20.5 × 10.3 × 7.1 cm and new right common iliac lymphadenopathy without metastases (Figure 1B). CT-guided re-biopsy and immunohistochemistry (IHC) demonstrated unclassified round cell sarcoma with INI1 loss (Supplemental Figures 1B–F). Unfortunately, the patient progressed during work-up, and on reevaluation resection was not recommended due to symptomatic progression rendering resection highly morbid.

PET/CT identified a bulky centrally necrotic right retroperitoneal soft tissue mass (SUVmax 21.4; liver SUVmean 2.3) extending into the right hemipelvis, a soft tissue nodule medial to the right psoas muscle (SUVmax 16.4), several non-enlarged FDG-avid paracaval lymph nodes (SUVmax 13.5), and faintly metabolic right external iliac and inguinal lymph nodes (Figure 1C). A single FDG-avid, non-enlarged left supraclavicular lymph node (SUVmax 8.6) was identified but not amenable to biopsy (stage IV: cT4N1M1; Figure 1D). Systemic first-line chemotherapy was recommended. No *EWSR1* fusion gene was identified by FISH (Abbott Molecular, Des Plaines, IL). Next-generation sequencing (NGS) of the primary tumor biopsy was negative for common gene fusions (*EWSR1, CIC*, or *BCOR*; Supplemental Table 1).

**Figure 2:**
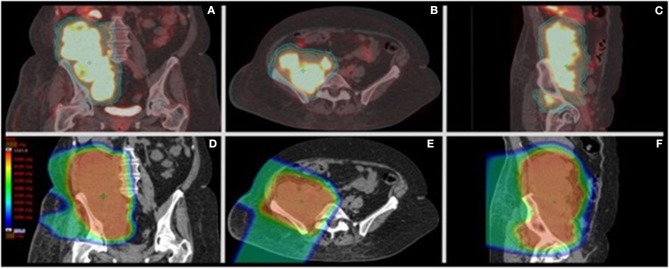
PET/CT of the primary retroperitoneal sarcoma with palliative proton radiotherapy plan dose distribution. **(A–C)** Representative coronal, axial, and sagittal images from the CT simulation for radiation treatment planning fused to the staging PET scan showing the clinical target volume (CTV-orange) and planning target volume (PTV-aqua) encompassing the right retroperitoneal FDG-avid disease. The CTV encompassed the FDG-avid sites of disease with a 0.5 cm expansion to create the PTV. **(D–F)** Representative coronal, axial, and sagittal images from the radiation treatment plan showing the dose color wash from right anterior and right posterior oblique beams. Dose color wash ranges from 2 CGE (dark blue) to >50 CGE (red) without significant exit dose beyond the 2 CGE isodose. Treatment was delivered using right anterior oblique and right posterior oblique passively scattered proton beams from a MEVION S250 unit.

Due to rapidly progressing symptomatic disease the patient was referred to radiation oncology. Palliative proton radiotherapy was recommended to the primary mass and adjacent FDG-contiguous lymphadenopathy [50 Cobalt Gray Equivalents (CGE)/25 fractions, 2 CGE/fraction; Mevion S250, Mevion Medical Systems, Inc., Littleton, MA] due to the tumor location, size, and close proximity to the right kidney and small bowel (Figure 2). The patient was informed that adjuvant systemic therapy was needed to treat the metastatic disease. Radiotherapy was delivered over 38 days without complications. Peripheral blood counts remained stable throughout treatment (absolute lymphocyte counts: 0.9–1.1 k/mm^3^, lower limit of normal: 1 k/mm^3^); the patient had mild, asymptomatic Grade 1 lymphopenia before and after radiotherapy (Supplemental Table 2).

**Figure 3:**
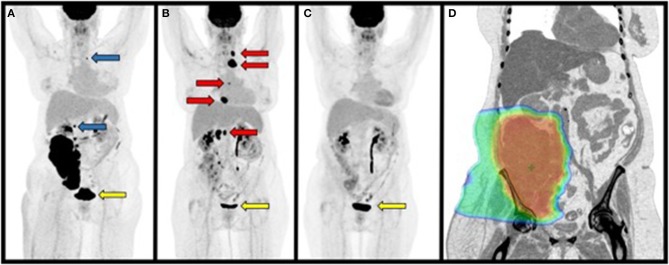
Maximum Intensity Projections (MIP) from PET/CT scans performed 1 month before, 1, and 5 months after radiation treatment. **(A)** PET/CT prior to radiation treatment demonstrating a large primary retroperitoneal mass, lesion superior to the mass at approximately T12 and left supraclavicular mass that were not included in the radiation treatment plan (blue arrows). **(B)** PET/CT performed 1.5 months after completing radiation treatment to the primary retroperitoneal mass demonstrating disease progression at sites outside of the radiation treatment field (red arrows). **(C)** PET/CT performed 5 months after completing radiation treatment demonstrating regression of disease at non-irradiated sites. **(D)** Representative inverted coronal image from the patient's CT simulation for radiation treatment showing radiation dose color wash covering the primary mass. Note that disease superior to the mass adjacent to the right kidney and left supraclavicular region were not included in this plan. Dose range: blue ≥ 2 CGE, green ≥ 25 CGE, red ≥ 50 CGE. Yellow arrows indicate physiologic uptake in the urinary bladder as in Figure 1.

PET/CT 1 month after radiation demonstrated disease progression at multiple sites outside of the radiotherapy field including the left supraclavicular lymph node (2.7 cm, previously 1 cm; SUVmax 18.1; liver SUVmean 2.6), T12-L1-adjacent retroperitoneal mass (1.4 cm; SUVmax 21.9, prior SUVmax 10.9), and new FDG-avid nodal metastases (RECIST 1.1; Figure 3B) (20). The primary RPS decreased in size and FDG-avidity (20.5 × 10.3 cm to 8.7 × 4.5 cm; SUVmax 20 to 7.1; Figure 3B). The patient reported significant improvement in right lower extremity edema, pain, range of motion, and performance status.

CT-guided needle biopsy of the left supraclavicular lymph node revealed metastatic disease from primary RPS (Supplemental Figure 2). The patient was offered palliative chemotherapy for disease progression on a clinical trial. NGS of the primary tumor re-biopsy identified a nonsense mutation in *SMARCB1* encoding INI1, confirmed by IHC loss of INI1 expression; no actionable mutation(s) outside of clinical trials were identified (FoundationOne CDx™, Foundation Medicine, Cambridge, MA; Supplemental Table 3). Tumor mutational burden and microsatellite stability were low, suggesting a lower probability of response to ICB (Supplemental Table 3).

The patient refused additional treatment and continued on close surveillance. PET/CT performed 5 months after radiation demonstrated near complete metabolic response of the biopsy-proven left supraclavicular metastasis (SUVmax 3.2, previously 18.1; liver SUVmean 2.5), and size reduction to <10 mm, with residual focal FDG avidity in the right retroperitoneum (SUVmax 4.4, previously 6.5) consistent with post-radiation changes vs. residual disease without other abnormal metabolic activity (Figure 3C).

**Figure 4:**
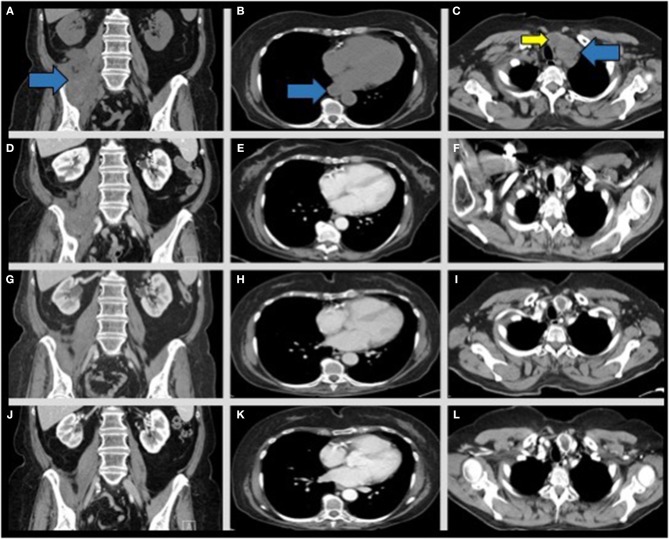
CT chest/abdomen/pelvis scans from post-radiation disease progression through continued interval follow up to monitor late responses to radiation therapy and to assess for recurrence. Representative coronal images from interval CT scans of the pelvic mass (left column- blue arrow), para-esophageal (middle column- blue arrow), and left biopsy-proven supraclavicular lymph node (right column- blue arrow) across several interval CT scans. **(A–C)** 1 month post-radiation without contrast. **(D–F)** 5 months post-radiation with contrast, **(G–I)** 7 months post-radiation with contrast, **(J–L)** 17 months post-radiation with contrast. Note imaging appearance of a stable, regressed right retroperitoneal mass (**D**, blue arrow), regression of the enlarged para-esophageal lymph node (**E**, blue arrow), and regression of the left supraclavicular lymph node noted at 5 months post-radiation (**F**, blue arrow). A previously identified enlarged left thyroid nodule is also present across the scans (**C,F,I,L**, yellow arrow) and is not consistent with metastatic disease.

The patient continued to improve, with interval imaging at 6, 10, 13, and 17 months post-radiotherapy demonstrating residual RPS/scar tissue and resolved metastases (Figure 4). Given the prolonged disease-free period without adjuvant therapy the patient agreed to PD-L1 assessment for ICB using nivolumab (28-8 pharmDx, PhenoPath; Seattle, Washington). PD-L1 expression was >/=1% for the primary RPS [tumor proportion score (TPS) 1–5] and metastatic left supraclavicular lymph node (TPS 1–10). CD4 and CD8 IHC on the pre-radiation RPS biopsy demonstrated TILs (CD4 10% positive, CD8 2% positive; 5:1 ratio) arranged in scattered nodules with patchy single-cell infiltration throughout, with similar results found for the non-irradiated left supraclavicular lymph node biopsied 1 month after completing radiation (CD4 10% positive, CD8 2% positive; 5:1 ratio; Supplemental Figures 2, 3).

The patient remains without evidence of new metastatic disease, completely regressed metastatic lesions, and nearly resolved primary RPS with residual scar tissue. She is nearly 2 years from initial diagnosis and ~1.5 years following proton radiotherapy. If her disease progresses she may receive chemotherapy or ICB based PD-L1 expression (21).

## Discussion

The abscopal effect (“*ab”*—position away from, “*scopus”*—target), was coined in 1953 by Dr. Mole as “an action at a distance from the irradiated volume but within the same organism,” and now describes regression of non-irradiated tumor lesions following radiotherapy (22). Abscopal reports are infrequent, with <50 reports from 1969 to 2014, occurring in immunogenic histologies (e.g., lymphoma, melanoma, renal cell carcinoma) following multimodality treatment (23, 24). Reports of abscopal effects have increased over the past decade, with many observed after combination treatment with radiotherapy and ICB targeting CTLA-4 or the PD-1/PD-L1 axis (25). Well-designed preclinical studies demonstrated the immunological dependence of radiotherapy-induced abscopal effects and potential for augmentation by ICB, wherein radiation serves as an *in situ* vaccination against tumor-associated antigens (TAAs); this effect appears to be suppressed at higher doses over 10 Gy per fraction (26–28). The biological mechanisms underlying radiation-induced immune responses have been extensively reviewed elsewhere and involve both innate and adaptive immune responses following radiation-induced double-stranded DNA damage in response to increased cytosolic levels of tumor cell DNA sufficient to drive increase tumor infiltration by antigen presenting cells that cross-present TAAs to CD8 T cells and adaptive anti-tumor immune responses (28, 29). These cascades are mediated by radiation-induced generation of Type I interferons, secretion of chemotactic ligands and cytokines, and upregulation of MHC I on tumor cells, resulting in increased tumor-infiltrating lymphocytes (TILs) (29). Photon-based radiation *in situ* vaccination is often insufficient to mediate abscopal responses due to suboptimal activation of these pathways, as well as multiple opposing mechanisms (e.g., selective enrichment of tumor infiltrating radio-resistant myeloid/monocytic inhibitory cells, radiation-induced PD-L1 upregulation, and immunosuppressive cytokine upregulation) within the tumor microenvironment that may be overcome by ICB (30–34).

Abscopal effects following radiation monotherapy provide unique opportunities to identify and optimize actionable variables of radiotherapy delivery crucial to promoting *in situ* vaccination. Effector lymphocytes mediate abscopal effects, and higher TILs in pre-treatment tumor specimens predicts OS (29, 35, 36). Yet it is known that post-treatment lymphopenia is associated with poor clinical outcomes and is correlated with radiation target volume and addition of chemotherapy (37–39). Radiation alone can induce volume-dependent lymphopenia via irradiation of lymphoid organs, bone marrow, and circulating lymphocytes (40). Radiation modality may also contribute to lymphopenia, as esophageal cancer patients treated with photons experienced more Grade 4 lymphopenia compared to those treated with protons, suggesting a dependence on total integral dose to lymphocyte-containing normal structures such as the vasculature (41). Lymphocytes are very radiosensitive, susceptible to apoptosis in response to doses as low as 1–2 Gy, with evidence indicating that activated T cells may be more radio-resistant (31, 32, 42). Circulating immune cells vary in radio-sensitivity; monocytes are considered radio-resistant *in vitro*, and radiation may skew the tumor infiltrating cell profile in favor of these suppressive cells following treatment (32). Our patient did not receive any chemotherapy that could have induced lymphopenia. Instead, she received protons to reduce dose to small bowel adjacent to her primary mass, as charged particles (i.e., protons, carbon ions) have different dose deposition profiles (e.g., Bragg peak) compared to photons, with little dose delivered beyond the target, that could potentially reduce toxicity (43). Proton therapy also had the potential ancillary benefit of significantly reducing radiation dose to the aorta, inferior vena cava, and draining lymph nodes, reducing radiation exposure to the circulating lymphocytes as well as reducing dose to the bone marrow (Figure 2 and Supplemental Figure 4) (44).

Evidence indicates that immunogenic cell death induced by protons is comparable to photons (45). Abscopal responses in metastatic patients treated with carbon ions have also been observed; however, to our knowledge, there have been no reports of abscopal responses in patients treated with proton radiotherapy (46). Preclinical work in murine models suggests elective irradiation of tumor-draining lymph nodes abrogates anti-tumor immune responses by altering chemokine expression and reducing intratumoral effector T cell infiltrates (47). Radiation also up-regulates specific gene products, generating putative tumor neoantigens that may further assist in promoting immune-mediated anti-tumor responses (48). Circulating lymphocytes and lymph nodes have been suggested to be considered an organ at risk to avoid lymphopenia during and after radiation (49). Photon-based radiation upregulates immunosuppressive products as well (e.g., PD-L1), that may antagonize radiation-promoted immune responses (33, 34). It is unclear if proton radiotherapy also induces PD-L1 expression, and warrants further investigation. Thus, in this case proton radiotherapy may have spared perturbation of draining lymph node microenvironments, and improved antigen presentation.

The patient's RPS had pretreatment CD4/CD8 infiltration similar to the non-irradiated metastatic lesion suggesting pre-treatment immunogenicity in the primary tumor (Supplemental Figure 3). Radiotherapy likely induced death of TILs in the primary, however the similar CD4/CD8 TIL ratio in the non-treated metastatic lesion suggests it may have provided a tumor antigen source and haven for primed effector cells after radiotherapy. The potential clinical significance of radiotherapy-primed effector cells was suggested by the results of a secondary analysis of KEYNOTE 001 in which receipt of prior palliative radiotherapy was associated with a statistically significant improvement in overall survival for metastatic non-small cell lung cancer patients treated with pembrolizumab, even though palliative radiotherapy is not expected to improve survival outcomes on its own (50). Results remained significant after stratifying for prior systemic therapy. In colorectal cancer, there is evidence that radiation increases tumor-specific immune responses against antigens such as survivin (51). The magnitude and duration of our patient's disease regression on interval imaging is impressive, and consistent with a clinical abscopal effect at multiple sites. Our patient did not receive first-line chemotherapy or experience treatment-related lymphopenia, with detectable TILs in the pre-treatment lesion. However, the immune contribution to this abscopal effect remains a correlative clinical observation without *a priori* knowledge of the specific tumor antigen(s) needed to monitor antigen-specific CD8 clonal expansion following radiotherapy but is feasible by prospective NGS analysis of infiltrating effector T cell receptor (TCR) diversity (52).

Prospective investigation of radiotherapy-primed abscopal effects for metastatic RPS could benefit from analyzing TCR diversity pre-and-post treatment to identify antigen-specific T cell clonal expansion elicited by radiotherapy that is usually insufficient to induce clinically meaningful responses. Radiation-induced antigen-specific responses may be potentiated by ICB, as suggested by the results of the KEYNOTE 001 secondary analysis (50). Our patient progressed at non-irradiated sites during and after radiotherapy, then had a delayed response post-treatment over several months, consistent with the abscopal effect (Figure 3). Whether this response was potentiated by the use of proton therapy to avoid excess radiation to circulating lymphocytes remains unclear but is hypothesis-generating (Supplemental Figure 4). It is noteworthy that our patient experienced a durable and complete response to therapy exceeding the median OS for similar RPS patients with nodal metastases at presentation (17, 18) (Figure 3).

Our patient had an unclassified round cell RPS, consisting of small round cells without gene rearrangement (*EWSR1, CIC*, or *BCOR* non-rearranged), a finding associated with poor prognosis (53). Molecular profiling using NGS identified a *SMARCB1* nonsense mutation with corresponding loss of INI1 expression by IHC (Supplemental Figure 1). INI1 is a known tumor suppressor, and INI loss is a validated marker for epithelioid sarcoma and malignant rhabdoid tumors. INI1 loss also occurs in other aggressive malignancies and is considered a poor prognostic factor, upregulating enhancer of zeste homolog 2 (EZH2), a histone-lysine *N*-methyltransferase that represses gene transcription (54). Inhibition of EZH2 using tazemetostat has shown promise in treating solid tumors with mutations of *SMARC* family genes, including epithelioid sarcoma (55). Interestingly, an abscopal effect was observed in a patient with a *SMARCB1*/INI1-deficient sacral chordoma treated on a Phase II study allowing multiple sequential biopsies before and after neoadjuvant tazemetostat and radiotherapy, documenting increases in pre-radiotherapy TILs (56). Elevated EZH2 levels correspond with radiation resistance, yet preclinical evidence indicates radiotherapy alone may be sufficient to reduce EZH2 protein expression *in vitro* and at the mRNA level *in vivo* via p53-mediated inhibition of E2F1 (57, 58). Whether radiation-induced inhibition of EZH2 in the setting of *SMARCB1*-mutated loss of INI1 occurs *in vivo* in humans to increase radio-sensitivity and promote abscopal effects remains to be proven.

## Conclusions

This case report represents, to the best of our knowledge, the first description of an abscopal effect for metastatic unresectable RPS following proton beam radiotherapy. Proton radiation of the primary RPS achieved improved sparing of draining lymph nodes, bone marrow, and the circulating blood volume with minimal impact seen on circulating lymphocyte counts during and after radiation treatment. Un-irradiated distant metastases regressed after treatment of the primary and in the absence of any systemic therapy, consistent with an abscopal effect. Molecular profiling using NGS identified a *SMARCB1* nonsense mutation and IHC identified loss of INI1 expression in the primary tumor that was also documented in a recent case report of the abscopal effect following EZH2 inhibition and radiotherapy for a sacral chordoma (56). Palliative radiotherapy for inoperable metastatic RPS may generate beneficial anti-tumor immune responses against un-irradiated sites of disease in selected patients. Limiting radiation doses to circulating blood volume to avoid radiotherapy-induced lymphopenia may augment radiation-induced immune responses promoted by post-treatment ICB, although additional research is needed. The profound abscopal response and putative mechanisms observed in this case report in metastatic RPS are hypothesis-generating. Future work is required to determine if proton radiotherapy, which could reduce radiation dose to circulating lymphocytes, may have an advantage in eliciting abscopal effects compared to standard radiotherapy.

## Data Availability

All datasets analyzed for this study are included in the manuscript and the Supplementary Files.

## Ethics Statement

Written informed consent was obtained from the individual(s) for the publication of any potentially identifiable images or data included in this article.

## Author Contributions

RB identified patient response, acquired patient data, wrote, and critically revised the manuscript. NS and JC acquired data, performed and interpreted pathology staining, and critically revised the manuscript. BF-V, CH, and JM critically revised manuscript and provided intellectual content. JG assisted with image interpretation and formatting and critically revised the manuscript. PO treating medical oncologist, acquired data, and critically revised the manuscript. BB treating radiation oncologist, identified patient response, acquired data, wrote and critically revised manuscript and provided intellectual content.

### Conflict of Interest Statement

JM reports consulting or advisory roles with Mevion outside of the submitted work. The remaining authors declare that the research was conducted in the absence of any commercial or financial relationships that could be construed as a potential conflict of interest.
