# Supplementary material for: Abscopal Effect Following Proton Beam Radiotherapy in a Patient With Inoperable Metastatic Retroperitoneal Sarcoma
Source: Front Oncol. 2019 Sep 26;9:922. doi: 10.3389/fonc.2019.00922 (PMC6775241; doi:10.3389/fonc.2019.00922)
Supplement: Supplementary file 1 [file Presentation_1.pptx]

## Slide 1
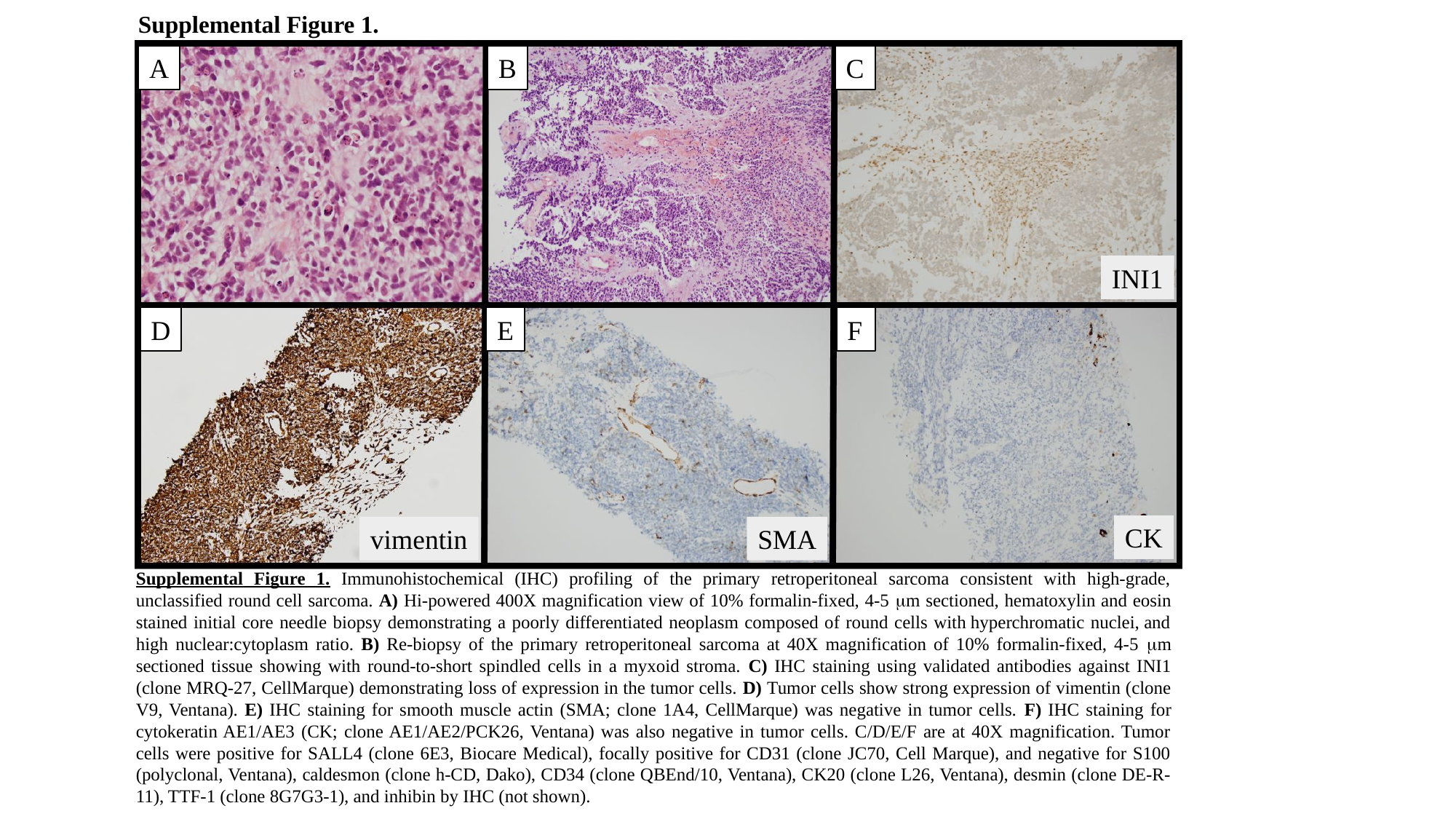

Supplemental Figure 1.
A
B
C
D
E
F
INI1
CK
vimentin
SMA
Supplemental Figure 1. Immunohistochemical (IHC) profiling of the primary retroperitoneal sarcoma consistent with high-grade, unclassified round cell sarcoma. A) Hi-powered 400X magnification view of 10% formalin-fixed, 4-5 μm sectioned, hematoxylin and eosin stained initial core needle biopsy demonstrating a poorly differentiated neoplasm composed of round cells with hyperchromatic nuclei, and high nuclear:cytoplasm ratio. B) Re-biopsy of the primary retroperitoneal sarcoma at 40X magnification of 10% formalin-fixed, 4-5 μm sectioned tissue showing with round-to-short spindled cells in a myxoid stroma. C) IHC staining using validated antibodies against INI1 (clone MRQ-27, CellMarque) demonstrating loss of expression in the tumor cells. D) Tumor cells show strong expression of vimentin (clone V9, Ventana). E) IHC staining for smooth muscle actin (SMA; clone 1A4, CellMarque) was negative in tumor cells. F) IHC staining for cytokeratin AE1/AE3 (CK; clone AE1/AE2/PCK26, Ventana) was also negative in tumor cells. C/D/E/F are at 40X magnification. Tumor cells were positive for SALL4 (clone 6E3, Biocare Medical), focally positive for CD31 (clone JC70, Cell Marque), and negative for S100 (polyclonal, Ventana), caldesmon (clone h-CD, Dako), CD34 (clone QBEnd/10, Ventana), CK20 (clone L26, Ventana), desmin (clone DE-R-11), TTF-1 (clone 8G7G3-1), and inhibin by IHC (not shown).

## Slide 2
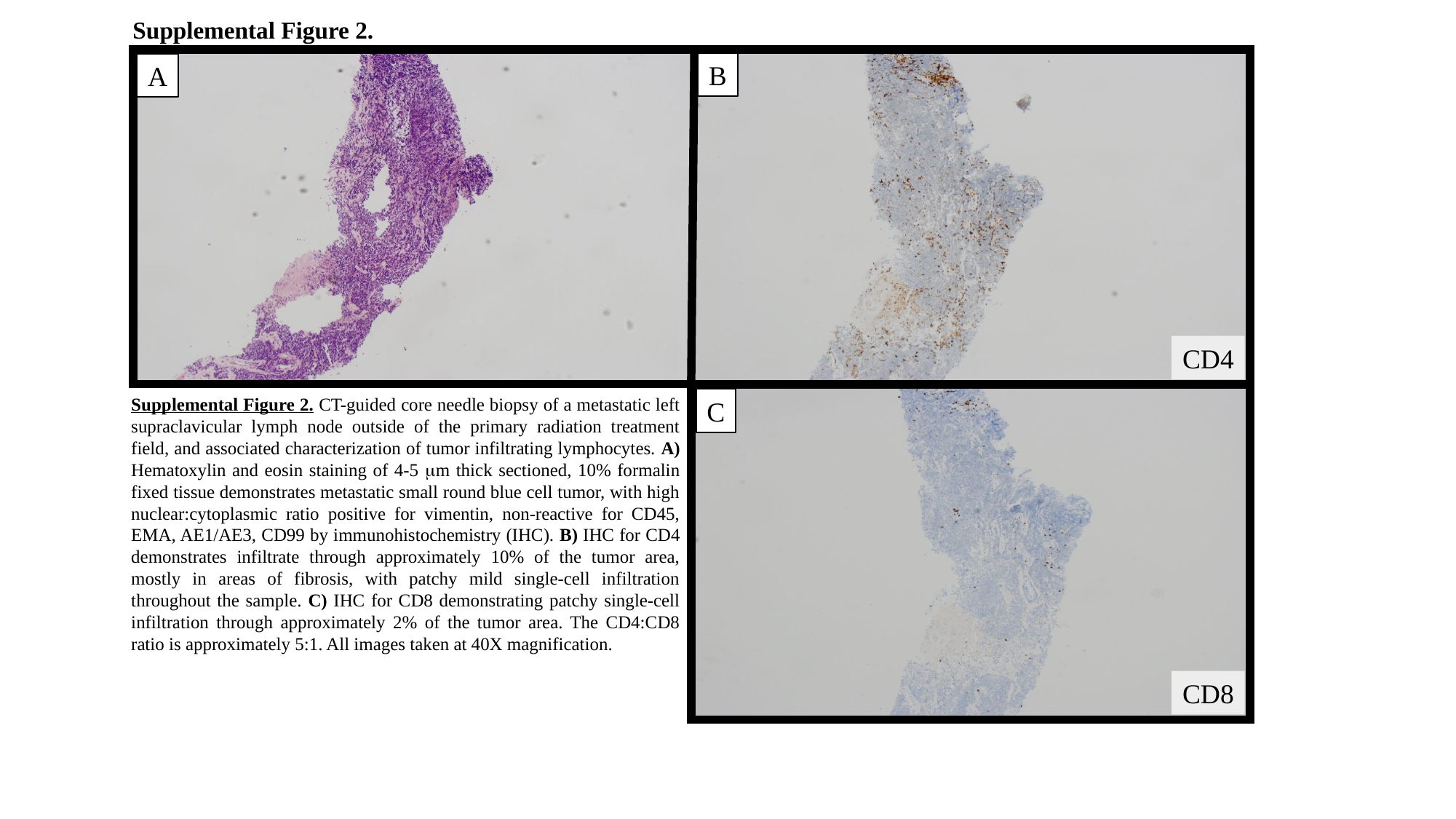

Supplemental Figure 2.
B
A
CD4
Supplemental Figure 2. CT-guided core needle biopsy of a metastatic left supraclavicular lymph node outside of the primary radiation treatment field, and associated characterization of tumor infiltrating lymphocytes. A) Hematoxylin and eosin staining of 4-5 μm thick sectioned, 10% formalin fixed tissue demonstrates metastatic small round blue cell tumor, with high nuclear:cytoplasmic ratio positive for vimentin, non-reactive for CD45, EMA, AE1/AE3, CD99 by immunohistochemistry (IHC). B) IHC for CD4 demonstrates infiltrate through approximately 10% of the tumor area, mostly in areas of fibrosis, with patchy mild single-cell infiltration throughout the sample. C) IHC for CD8 demonstrating patchy single-cell infiltration through approximately 2% of the tumor area. The CD4:CD8 ratio is approximately 5:1. All images taken at 40X magnification.
C
CD8

## Slide 3
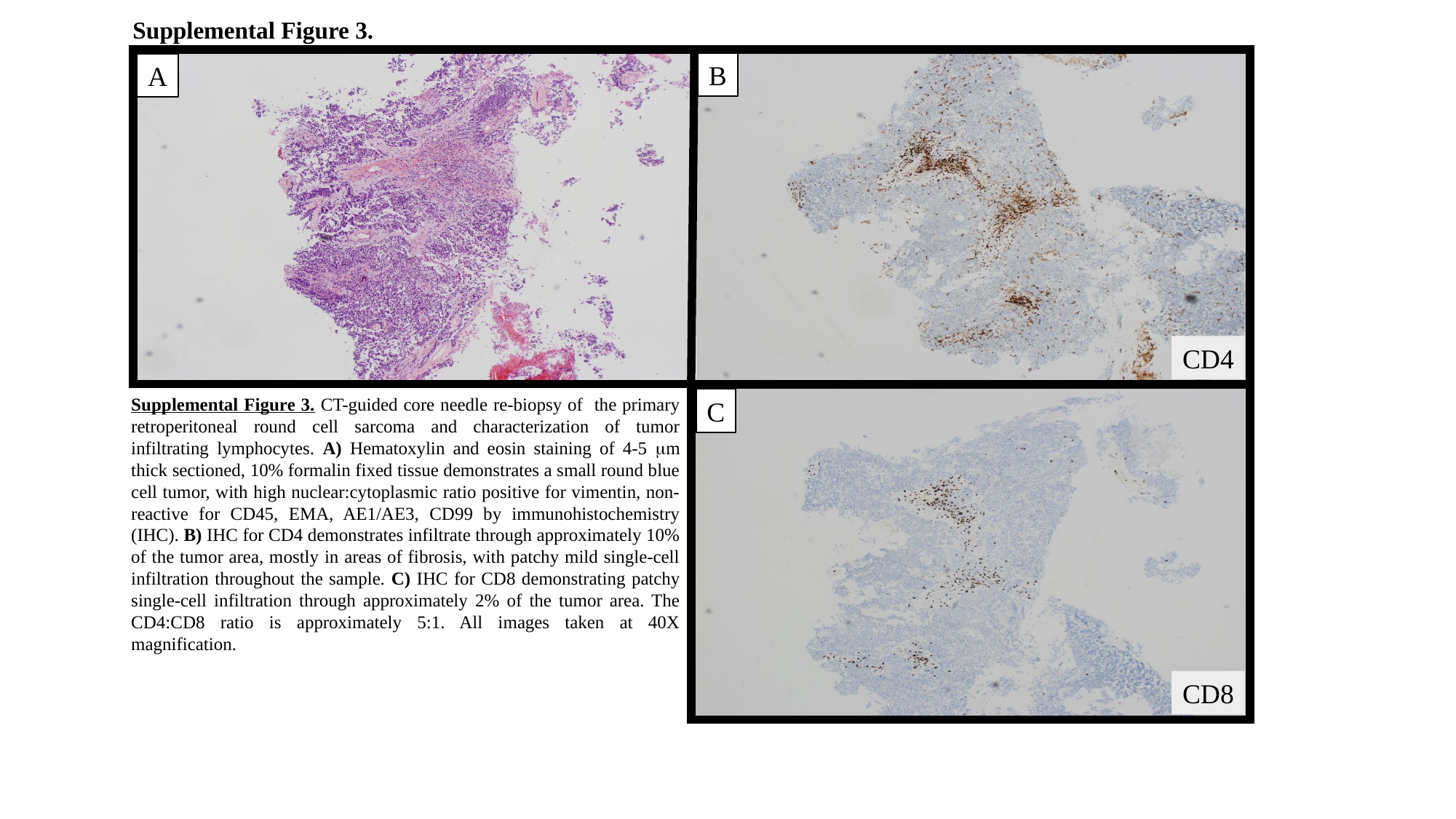

Supplemental Figure 3.
B
A
CD4
Supplemental Figure 3. CT-guided core needle re-biopsy of the primary retroperitoneal round cell sarcoma and characterization of tumor infiltrating lymphocytes. A) Hematoxylin and eosin staining of 4-5 μm thick sectioned, 10% formalin fixed tissue demonstrates a small round blue cell tumor, with high nuclear:cytoplasmic ratio positive for vimentin, non-reactive for CD45, EMA, AE1/AE3, CD99 by immunohistochemistry (IHC). B) IHC for CD4 demonstrates infiltrate through approximately 10% of the tumor area, mostly in areas of fibrosis, with patchy mild single-cell infiltration throughout the sample. C) IHC for CD8 demonstrating patchy single-cell infiltration through approximately 2% of the tumor area. The CD4:CD8 ratio is approximately 5:1. All images taken at 40X magnification.
C
CD8

## Slide 4
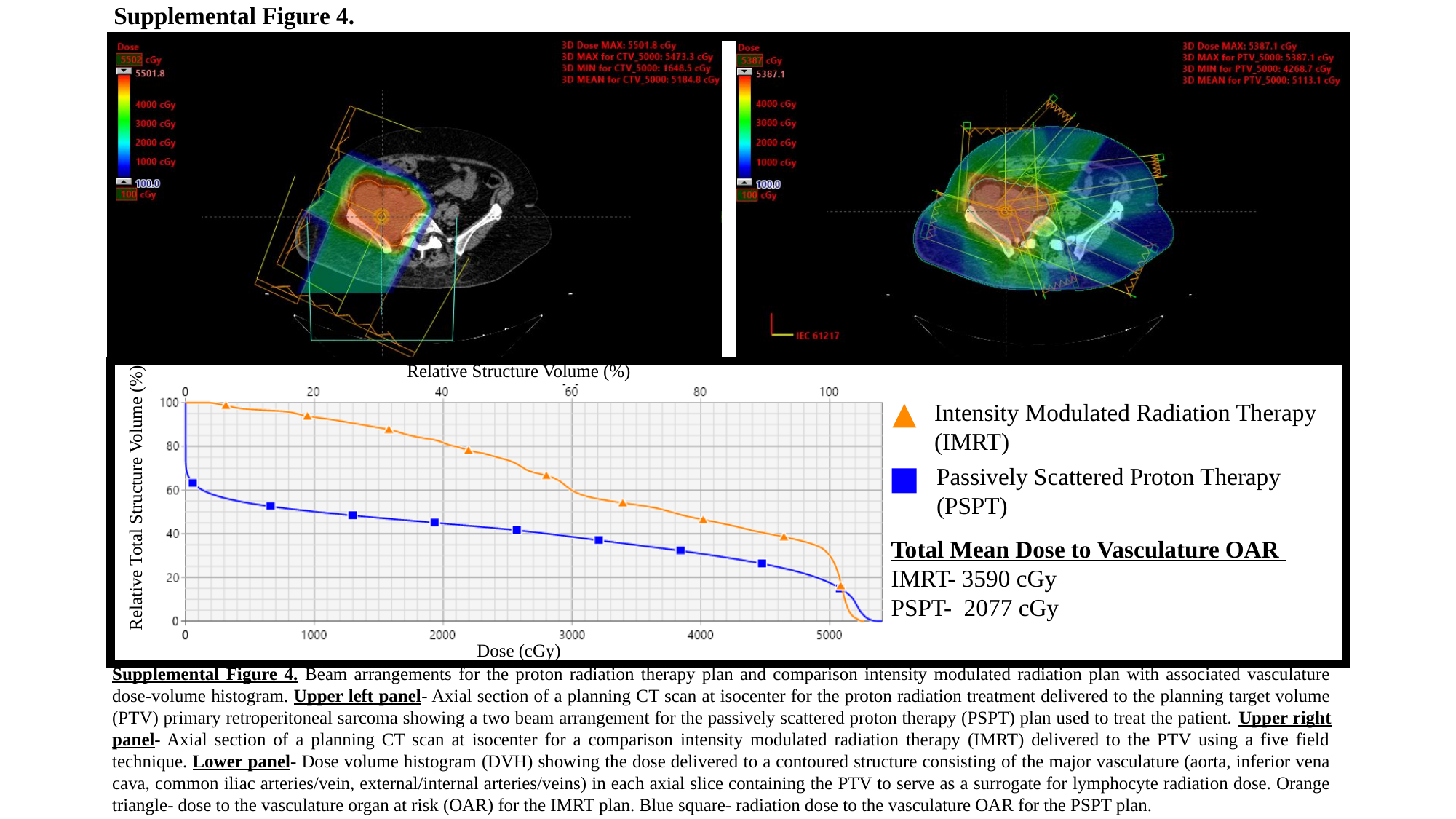

Supplemental Figure 4.
Relative Structure Volume (%)
Intensity Modulated Radiation Therapy (IMRT)
Passively Scattered Proton Therapy (PSPT)
Relative Total Structure Volume (%)
Total Mean Dose to Vasculature OAR
IMRT- 3590 cGy
PSPT- 2077 cGy
Dose (cGy)
Supplemental Figure 4. Beam arrangements for the proton radiation therapy plan and comparison intensity modulated radiation plan with associated vasculature dose-volume histogram. Upper left panel- Axial section of a planning CT scan at isocenter for the proton radiation treatment delivered to the planning target volume (PTV) primary retroperitoneal sarcoma showing a two beam arrangement for the passively scattered proton therapy (PSPT) plan used to treat the patient. Upper right panel- Axial section of a planning CT scan at isocenter for a comparison intensity modulated radiation therapy (IMRT) delivered to the PTV using a five field technique. Lower panel- Dose volume histogram (DVH) showing the dose delivered to a contoured structure consisting of the major vasculature (aorta, inferior vena cava, common iliac arteries/vein, external/internal arteries/veins) in each axial slice containing the PTV to serve as a surrogate for lymphocyte radiation dose. Orange triangle- dose to the vasculature organ at risk (OAR) for the IMRT plan. Blue square- radiation dose to the vasculature OAR for the PSPT plan.

## Slide 5
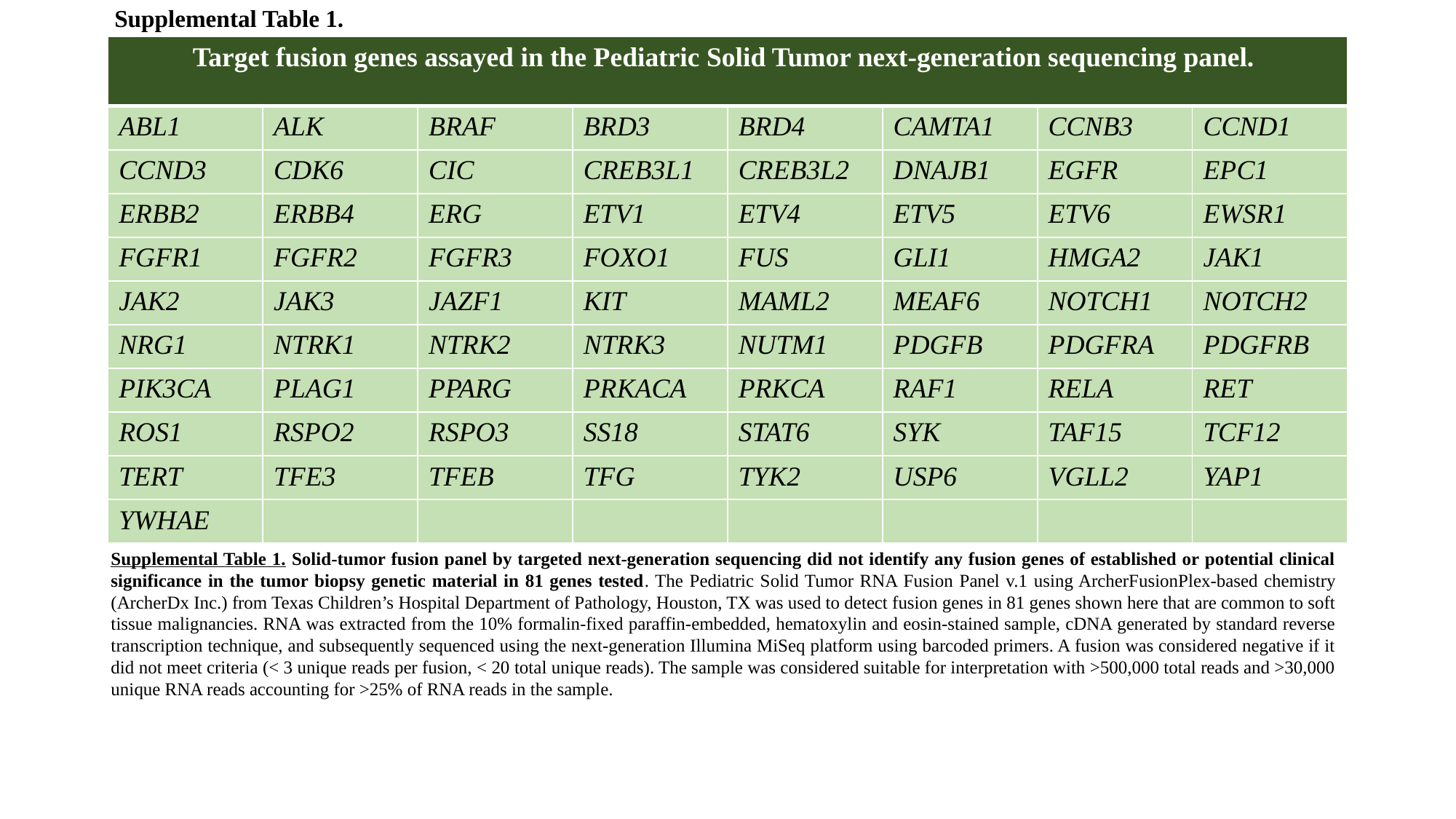

Supplemental Table 1.
| Target fusion genes assayed in the Pediatric Solid Tumor next-generation sequencing panel. | | | | | | | |
| --- | --- | --- | --- | --- | --- | --- | --- |
| ABL1 | ALK | BRAF | BRD3 | BRD4 | CAMTA1 | CCNB3 | CCND1 |
| CCND3 | CDK6 | CIC | CREB3L1 | CREB3L2 | DNAJB1 | EGFR | EPC1 |
| ERBB2 | ERBB4 | ERG | ETV1 | ETV4 | ETV5 | ETV6 | EWSR1 |
| FGFR1 | FGFR2 | FGFR3 | FOXO1 | FUS | GLI1 | HMGA2 | JAK1 |
| JAK2 | JAK3 | JAZF1 | KIT | MAML2 | MEAF6 | NOTCH1 | NOTCH2 |
| NRG1 | NTRK1 | NTRK2 | NTRK3 | NUTM1 | PDGFB | PDGFRA | PDGFRB |
| PIK3CA | PLAG1 | PPARG | PRKACA | PRKCA | RAF1 | RELA | RET |
| ROS1 | RSPO2 | RSPO3 | SS18 | STAT6 | SYK | TAF15 | TCF12 |
| TERT | TFE3 | TFEB | TFG | TYK2 | USP6 | VGLL2 | YAP1 |
| YWHAE | | | | | | | |
Supplemental Table 1. Solid-tumor fusion panel by targeted next-generation sequencing did not identify any fusion genes of established or potential clinical significance in the tumor biopsy genetic material in 81 genes tested. The Pediatric Solid Tumor RNA Fusion Panel v.1 using ArcherFusionPlex-based chemistry (ArcherDx Inc.) from Texas Children’s Hospital Department of Pathology, Houston, TX was used to detect fusion genes in 81 genes shown here that are common to soft tissue malignancies. RNA was extracted from the 10% formalin-fixed paraffin-embedded, hematoxylin and eosin-stained sample, cDNA generated by standard reverse transcription technique, and subsequently sequenced using the next-generation Illumina MiSeq platform using barcoded primers. A fusion was considered negative if it did not meet criteria (< 3 unique reads per fusion, < 20 total unique reads). The sample was considered suitable for interpretation with >500,000 total reads and >30,000 unique RNA reads accounting for >25% of RNA reads in the sample.

## Slide 6
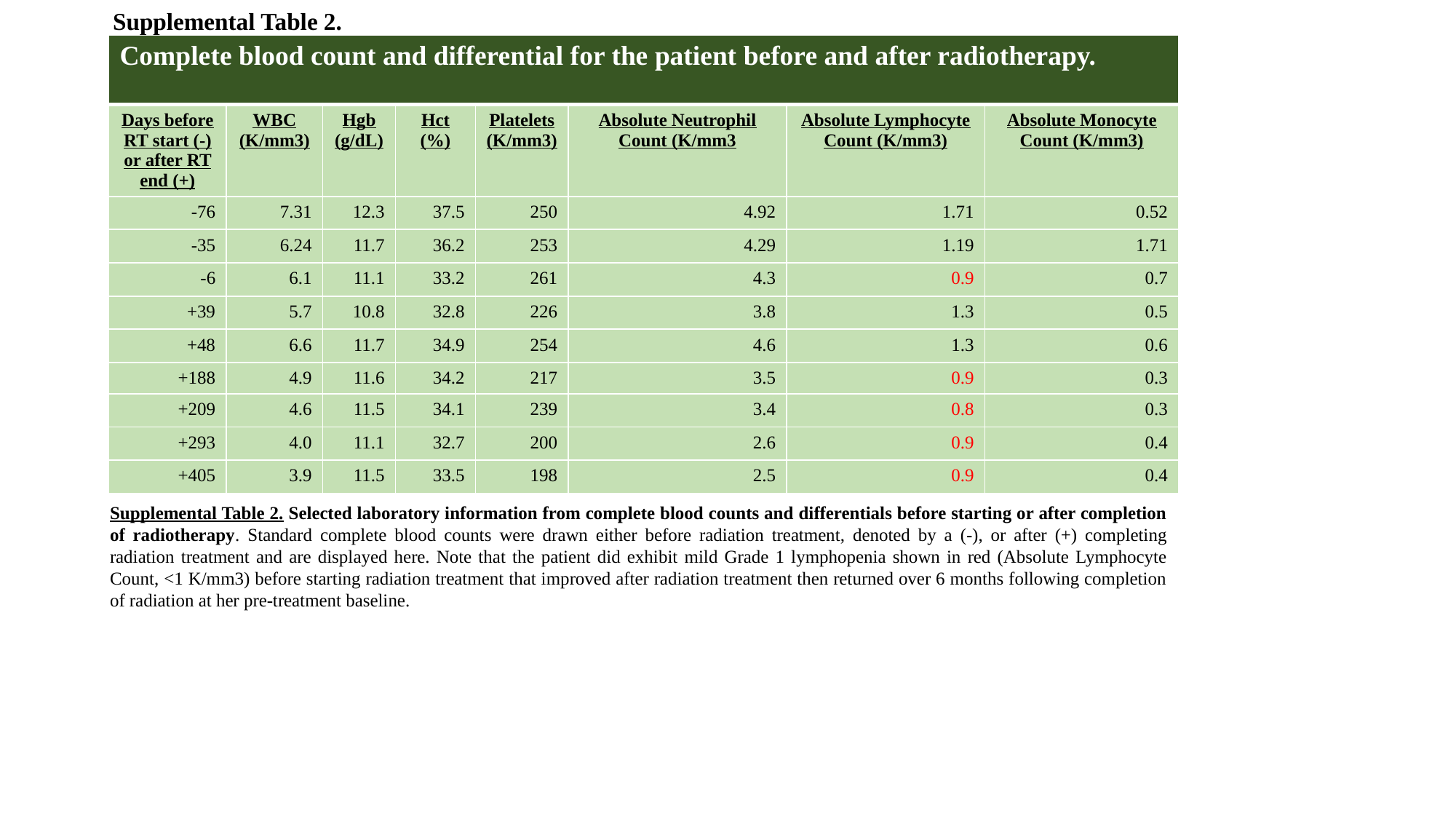

Supplemental Table 2.
| Complete blood count and differential for the patient before and after radiotherapy. | | | | | | | |
| --- | --- | --- | --- | --- | --- | --- | --- |
| Days before RT start (-) or after RT end (+) | WBC (K/mm3) | Hgb (g/dL) | Hct (%) | Platelets (K/mm3) | Absolute Neutrophil Count (K/mm3 | Absolute Lymphocyte Count (K/mm3) | Absolute Monocyte Count (K/mm3) |
| -76 | 7.31 | 12.3 | 37.5 | 250 | 4.92 | 1.71 | 0.52 |
| -35 | 6.24 | 11.7 | 36.2 | 253 | 4.29 | 1.19 | 1.71 |
| -6 | 6.1 | 11.1 | 33.2 | 261 | 4.3 | 0.9 | 0.7 |
| +39 | 5.7 | 10.8 | 32.8 | 226 | 3.8 | 1.3 | 0.5 |
| +48 | 6.6 | 11.7 | 34.9 | 254 | 4.6 | 1.3 | 0.6 |
| +188 | 4.9 | 11.6 | 34.2 | 217 | 3.5 | 0.9 | 0.3 |
| +209 | 4.6 | 11.5 | 34.1 | 239 | 3.4 | 0.8 | 0.3 |
| +293 | 4.0 | 11.1 | 32.7 | 200 | 2.6 | 0.9 | 0.4 |
| +405 | 3.9 | 11.5 | 33.5 | 198 | 2.5 | 0.9 | 0.4 |
Supplemental Table 2. Selected laboratory information from complete blood counts and differentials before starting or after completion of radiotherapy. Standard complete blood counts were drawn either before radiation treatment, denoted by a (-), or after (+) completing radiation treatment and are displayed here. Note that the patient did exhibit mild Grade 1 lymphopenia shown in red (Absolute Lymphocyte Count, <1 K/mm3) before starting radiation treatment that improved after radiation treatment then returned over 6 months following completion of radiation at her pre-treatment baseline.

## Slide 7
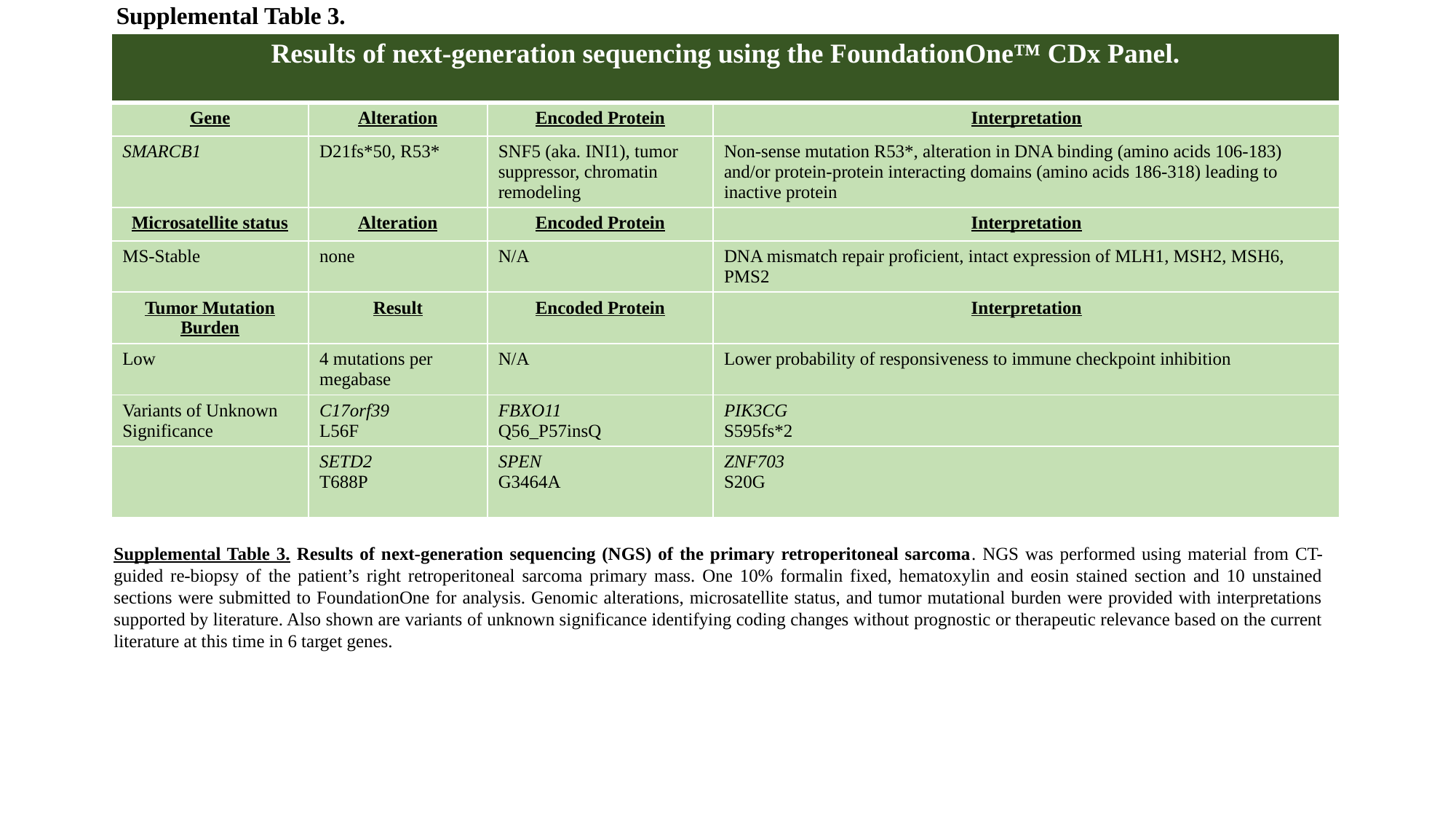

Supplemental Table 3.
| Results of next-generation sequencing using the FoundationOne™ CDx Panel. | | | |
| --- | --- | --- | --- |
| Gene | Alteration | Encoded Protein | Interpretation |
| SMARCB1 | D21fs\*50, R53\* | SNF5 (aka. INI1), tumor suppressor, chromatin remodeling | Non-sense mutation R53\*, alteration in DNA binding (amino acids 106-183) and/or protein-protein interacting domains (amino acids 186-318) leading to inactive protein |
| Microsatellite status | Alteration | Encoded Protein | Interpretation |
| MS-Stable | none | N/A | DNA mismatch repair proficient, intact expression of MLH1, MSH2, MSH6, PMS2 |
| Tumor Mutation Burden | Result | Encoded Protein | Interpretation |
| Low | 4 mutations per megabase | N/A | Lower probability of responsiveness to immune checkpoint inhibition |
| Variants of Unknown Significance | C17orf39 L56F | FBXO11 Q56\_P57insQ | PIK3CG S595fs\*2 |
| | SETD2 T688P | SPEN G3464A | ZNF703 S20G |
Supplemental Table 3. Results of next-generation sequencing (NGS) of the primary retroperitoneal sarcoma. NGS was performed using material from CT-guided re-biopsy of the patient’s right retroperitoneal sarcoma primary mass. One 10% formalin fixed, hematoxylin and eosin stained section and 10 unstained sections were submitted to FoundationOne for analysis. Genomic alterations, microsatellite status, and tumor mutational burden were provided with interpretations supported by literature. Also shown are variants of unknown significance identifying coding changes without prognostic or therapeutic relevance based on the current literature at this time in 6 target genes.
